# Supplementary figures and images for: Integrated transcriptomics and metabolomics reveal signatures of lipid metabolism dysregulation in HepaRG liver cells exposed to PCB 126
Source: Arch Toxicol. 2018 Jun 14;92(8):2533–47. doi: 10.1007/s00204-018-2235-7 (PMC6063328; doi:10.1007/s00204-018-2235-7)

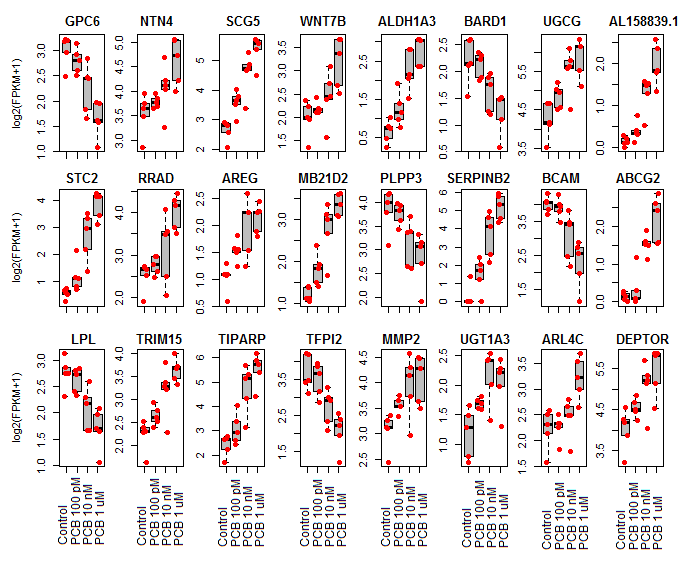

Supplement: Supplementary file 2 — Box plot of transcriptome changes associated with the exposure to PCB 126 in HepaRG cells. All the transcript displayed have their levels significantly altered (q < 0.05). Most of the changes caused by the PCB treatment were dose dependent. (TIF 100 KB) [file 204_2018_2235_MOESM2_ESM.tif]

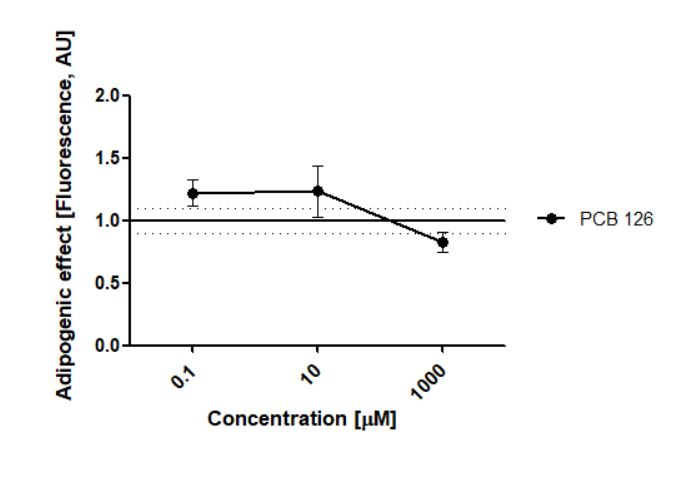

Supplement: Supplementary file 5 — Measurement of lipid accumulation in HepaRG cells treated with PCB 126. The absence of phenotypic alterations at the timepoint chosen in this study were verified using the AdipoRed assay. This assay was performed in triplicate according to manufacturer’s recommendations. (JPEG 28 KB) [file 204_2018_2235_MOESM5_ESM.jpg]
